# Rapid Identification of Lineage and Drug Resistance in Clinical Samples of *Mycobacterium tuberculosis*

**DOI:** 10.3390/microorganisms11061467

**Published:** 2023-05-31

**Authors:** Jéssica Comín, Jesús Viñuelas, Carmen Lafoz, Alberto Cebollada, Daniel Ibarz, María-José Iglesias, Sofía Samper

**Affiliations:** 1 Instituto Aragonés de Ciencias de la Salud, C/de San Juan Bosco 13, 50009 Zaragoza, Spain; 2 Servicio de Microbiología, Hospital Universitario Miguel Servet, Paseo Isabel la Católica 1-3, 50009 Zaragoza, Spain; jvinuelas@aragon.es; 3 Grupo de Estudio de Infecciones por Micobacterias (GEIM), Sociedad Española de Enfermedades Infecciosas y Microbiología Clínica, C/Agustín de Bentacourt, No. 13, 28003 Madrid, Spain; 4 Servicio General de Apoyo a la Investigación, Servicio de Análisis Microbiológico, Universidad de Zaragoza, C/Pedro Cerbuna 12, 50009 Zaragoza, Spain; clafoz@unizar.es; 5 Unidad de Biocomputación, Instituto Aragonés de Ciencias de la Salud, C/de San Juan Bosco 13, 50009 Zaragoza, Spain; acebolladaso.iacs@aragon.es; 6 Grupo de Genética de Micobacterias, Facultad de Medicina, Universidad de Zaragoza, C/Domingo Miral S/N, 50009 Zaragoza, Spain; dibarz@unizar.es (D.I.); iglesias@unizar.es (M.-J.I.); 7 Fundación IIS Aragón, C/de San Juan Bosco 13, 50009 Zaragoza, Spain; 8 CIBER de Enfermedades Respiratorias, Av. Monforte de Lemos 3-5, Pabellón 11, Planta 0, 28029 Madrid, Spain

**Keywords:** tuberculosis, antimicrobial resistance, AmpliSeq technology, genomics, tuberculosis diagnosis

## Abstract

Background: *Mycobacterium tuberculosis* is a slow-growing bacterium, which could delay its diagnosis and, therefore, promote the spread of the disease. Whole-genome sequencing allows us to obtain the complete drug-resistance profile of the strain; however, bacterial cultivation of clinical samples, along with complex processing, is required. Methods: In this work, we explore AmpliSeq, an amplicon-based enrichment method for preparing libraries for targeted next-generation sequencing, to identify lineage and drug resistance directly from clinical samples. Results: In our study, 111 clinical samples were tested. The lineage was identified in 100% of the culture-derived samples (52/52), in 95% of the smear (BK)-positive clinical samples (38/40) and in 42.1% of the BK-negative clinical samples (8/19). The drug-resistance profile was accurately identified in all but 11 samples, in which some phenotypic and genotypic discrepancies were found. In this respect, our panels were not exact in the detection of streptomycin resistance for isolates derived from clinical samples, as an extremely high number of SNPs in the *rrs* and *rrl* genes were detected due to cross-contamination. Conclusion: This technique has demonstrated high sensitivity in obtaining the drug-resistance profile of the isolates, as even those samples with DNA concentrations below the detection limit of Qubit produced a result. AmpliSeq technology is cheaper than whole-genome sequencing, easy to perform by laboratory technicians and applicable to any microorganism using the Ion Torrent platform.

## 1. Introduction

Tuberculosis is caused by *Mycobacterium tuberculosis*, a slow-growing bacterium that requires two to four weeks to grow in culture, which, along with the need for drug susceptibility tests (DSTs), considerably delays diagnosis. Despite the availability of new drugs, the management of drug-resistant TB is further compounded by their high cost, long duration of treatment and higher toxicity. In an attempt to avoid resistance, both acquired and transmitted, it is necessary to join efforts in diagnosing possible existing resistance as soon as possible. Some rapid tests to detect *M. tuberculosis* and drug resistance are available; nevertheless, the number of mutations and examples of resistance detected by them are limited, especially for second-line drugs and for new drugs such as linezolid or bedaquiline. DNA can be extracted from liquid-medium mycobacterial growth indicator tubes [1] or directly from clinical samples [2,3,4,5]. At present, whole-genome sequencing (WGS) has become affordable; it provides the most exhaustive information about a strain’s drug-resistance profile [6]. In this way, the time to diagnosis and the optimisation of the treatment are considerably reduced from weeks to days. However, some difficulties are encountered when sequencing samples with low DNA concentrations. The knowledge provided by WGS allows us to focus on specific targets, diminishing the amount of unnecessary data and rendering the analysis easier. In particular, Ion AmpliSeq™ technology can design a multiplex PCR amplifying hundreds of sites of interest. The amount of DNA required for AmpliSeq is lower than for WGS, which improves the sensitivity. In addition, it is cheaper than WGS, as the sequencing is targeted instead of being on a whole-genome level, requiring fewer reads per sample.

The objective of this work was to test the efficiency of AmpliSeq and the quality of the data obtained regarding lineage identification and drug-resistance profile, using DNA extracted directly from clinical samples of patients infected with *M. tuberculosis*.

## 2. Materials and Methods

### 2.1. Set-Up of a DNA Extraction Method from Clinical Samples

**Figure 1:**
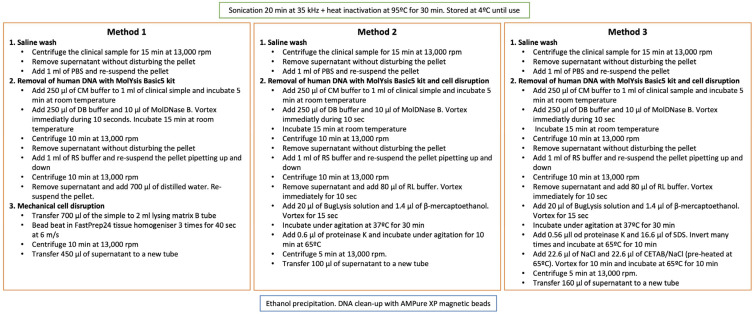
Description of the three protocols tested for DNA extraction directly from clinical samples. Samples containing more than 1 mL were worked in different vials and the pellets were collected together after the saline washing step. Method 1 was described by Votintseva et al. [1,2].

Three DNA extraction methods from clinical samples were tested (Figure 1). Before DNA extraction, the clinical samples were sonicated and heat-inactivated. Extracted DNA was cleaned using magnetic beads (AMPure XP). Method 1, based on mechanical cell disruption, was previously described [1]. Method 2 used chemical disruption using the buffers and enzymes provided by the MolYsis Basic5 kit (Molzym GmbH & Co. KG, Bremen, Germany) following the manufacturer’s instructions. For Method 3, the human DNA was also eliminated using the MolYsis Basic5 kit and a different chemical cell disruption method was used, based on NaCl/CTAB [7], the same used for extracting DNA from bacterial cultures. For testing the efficiency of the extraction, we used a VIASURE *Mycobacterium tuberculosis* complex Real Time PCR Detection kit (CERTEST Biotec S.L., Zaragoza, Spain).

### 2.2. Samples Analysed by AmpliSeq Technology

Since 2004, a surveillance protocol has been carried out in Aragon, Spain. As part of this protocol, all *M. tuberculosis* isolates are genotyped by IS*6110*-RFLP and spoligotyping. DSTs are performed by microbiologists in clinical laboratories. The 111 samples analysed in this study are detailed in Table S2. First, a collection of DNA was chosen from clinical strains, including different lineages and others with an undefined spoligotype, to classify them using AmpliSeq. Second, available retrospective samples taken for diagnosis stored at −80 °C were used, both smear-positive and -negative, but initially, all culture-positive sputum samples (54), biopsies (5), bronchoalveolar lavages (BAL) (3), bronchial aspirates (6), pleural fluids (4) and others (3) were collected and stored during the period 2018–2022 in the Microbiology Service of Miguel Servet Hospital in Zaragoza, Aragon, Spain. *M. tuberculosis* DNA was extracted from the samples and a standard PCR amplifying a 531 bp-region of the *dnaA* gene before AmpliSeq (dnaAj-F: CAATCGACAAAGCGCTGGC; dnaAj-R: TGGGGTGTGTGTTGGGT) was performed to confirm the presence of *M. tuberculosis* DNA. A positive result was obtained in 42 of the 75 samples (38 sputum, 2 biopsies, 1 aspirate and 1 pleural fluid) (Table 1). Seventeen samples with a negative PCR result were included to test the sensitivity of the technique, three with a positive BK result and fourteen (four sputum, two BAL samples, four bronchial aspirates, two pleural fluids, one biopsy and one adeno puncture) with a negative BK result. In addition, we included another 52 DNA extracts from bacterial cultures to compare the quality of the lineage and drug-resistance results to the DNA obtained directly from clinical samples. A total of 111 samples were included in the study. The DNA concentration was quantified using Qubit. All samples remained anonymous, and no patient data were handled. Our regional ethical committee (Comité de Ética de la Investigación de la Comunidad Autónoma de Aragón, Record No. 20/2018) approved the methodology, as detailed in the 18/0336 project.

### 2.3. AmpliSeq Technology

For target amplifications, two different AmpliSeq panels of primers were designed for lineage and drug-resistance identification using the Ion AmpliSeq Designer tool from Thermo Fisher Scientific. The first version (IAD88259) was designed based on the Ion AmpliSeq™ TB Research Panel developed for the *M. tuberculosis* drug-resistance diagnosis, adding new targets for a wider resistance profile and to provide lineage information for the *M. tuberculosis* complex. There were 29 targets, with 210 amplicons in a range of 125–375 bp covering approximately 20 kb. Two years later, a new version of the panel (IAD137392) was developed in order to complement the former version with updated targets. It included 68 targets, with 268 amplicons in a range of 125–375 bp and a coverage of 98.13%. The estimation of the total number of bases covered by the amplicons in the design was 52.65 kb. A complete list of the targets included in both panels can be found in Table S1. Fifty-five of the samples were analysed using the first version of the kit and fifty-six samples with the second version. The library preparation was carried out in the Ion Chef system and quantification was performed using the Ion Library TaqMan quantitation kit (Thermo Fisher Scientific, Waltham, MA, USA). Out of 111 samples, 55 were analysed using the first version of the kit (IAD88259) and 56 using the second version (IAD137392). The massive sequencing process was carried out in the IonGene Studio^TM^ S5 System using Ion 530^TM^ Chip. The sequences obtained were mapped against the reference strain H37Rv (NC_000962.3) and a coverage analysis was performed, followed by a variant calling.

### 2.4. Bioinformatic Analysis

A pipeline in R software was developed to obtain an automatic result. In this way, the mutations of interest were marked with their significance (Table S3.1) among the variants obtained, and automatically, the variants detected in the different samples were associated (i.e., Haarlem family or rifampicin resistance). For drug resistance, the variants associated with resistance in the catalogue of mutations in *M. tuberculosis* published by the World Health Organization (https://www.who.int/publications/i/item/9789240028173, accessed on 3 January 2022) and the PhyResSE website were considered [8].

## 3. Results

### 3.1. Set-Up of a DNA Extraction Method from Clinical Samples

The first step was to achieve efficient DNA extraction of good quality, as many clinical samples had a low bacterial load. Three different protocols were tested with two clinical samples (Figure 1). All of them were based on ethanol precipitation of the DNA. In order to test which method produced the highest yield, we extracted DNA from two clinical samples using the three methods and we carried out a real-time PCR using one VIASURE *M. tuberculosis* complex diagnostic kit. The PCR revealed that Method 1 obtained the highest yield in the two clinical samples tested and was chosen for extracting the DNA from the remaining clinical samples. The amount of DNA obtained in many of the clinical samples was below the detection limit of Qubit, but we included them in the study to test the sensitivity of the AmpliSeq technique, as some of those samples had been amplified by conventional PCR and served as controls for the DNA extraction (Table 1).

### 3.2. AmpliSeq Results

AmpliSeq results for the 111 samples analysed are shown in Table S2, comparing the DST and spoligotyping results to the AmpliSeq ones. The genomic lineage was correctly assigned in all the samples derived from bacterial culture. Regarding the clinical samples, the lineage was identified in 95% of the BK-positive samples (38/40) and in 42.1% of the BK-negative samples (8/19). In this sense, AmpliSeq provided information for some strains with an unknown or undetermined spoligo-family, complementing their genotype (Table S2).

The drug-resistance genotypes were correctly identified in 90.1% of the samples, according to the DST information of the strains obtained in the clinical laboratory. Moreover, additional SNPs were found in the RNA polymerase genes (*rpoB* and *rpoC*), not associated with resistance. Among the 17 rifampicin-resistant strains analysed, 9 harboured an additional mutation in *rpoB,* not related with a resistant phenotype (52.9%). Additionally, 7 out of the 12 (58.3%) rifampicin-resistant strains analysed with the second version of the panel, IAD137392, which added the *rpoC* gene as a target, harboured 1 or more mutations in the *rpoC* gene. Furthermore, fluoroquinolone resistance was unexpectedly identified for two susceptible samples, 658 and 659 (both belonging to the same patient), as second-line antibiotic tests are not routinely performed when the strains show susceptibility to the first-line drugs. On the other hand, some discrepancies were found among the genotype and phenotype drug-resistance profiles in 11 samples (Table S2). Sample 120 was streptomycin-resistant in the DST but no mutation in the *rrs* or *rpsL* genes was detected. Sample MTB-21 has a mutation in *embB* (Gln497Arg) described as conferring resistance, but the strain was susceptible to ethambutol in the DST. Sample MTB-12 harboured a mutation in the *pncA* gene (Gly17Ser) described as a confident SNP on the PhyResSE website, but the strain was pyrazinamide-susceptible in the DST. On the other hand, samples 537 and MTB-19 were pyrazinamide-resistant in the DST but no mutation in *pncA* was found in AmpliSeq. Sample MTB-22 was also pyrazinamide-resistant in the DST and it harboured an SNP in *pncA* (Lys48Asn) but was not described as conferring resistance. This sample was also streptomycin-resistant in the DST but no mutation was found in the *rpsL* gene (the *rrs* and *gid* genes were not included in the first version of the panel, so mutations there could not be discarded in this sample). Sample 942 was isoniazid-resistant in the DST and no mutations were found in AmpliSeq; however, after revising the bam files, we noticed that the reads were of poor quality and, thus, unreliable. Sample MTB-10 presented amikacin resistance in the DST, but the only mutation found with AmpliSeq in *rrs* (point 1,472,751) was described as conferring streptomycin resistance, not amikacin resistance. Sample MTB-14 was ethionamide-resistant in the DST and no mutation was found in the *inhA* gene by AmpliSeq (the *ethA* gene is not included in the panels as such a mutation there cannot be discarded). Sample MTB-16, resistant to isoniazid in the DST, only harboured a mutation in *katG* (Ser315Arg), not described as conferring resistance. Similarly, sample MTB-20 was streptomycin-resistant in the DST and the mutation found by AmpliSeq (*rpsL* Lys88Thr) was not described as conferring resistance. We found that 26 out of the 38 clinical samples analysed with the second version of the panel showed the *rrs* gene erroneously sequenced, with some regions of the gene containing a high number of SNPs. Despite this, none of the strains were identified as streptomycin-resistant.

Among the clinical samples with a low concentration in Qubit, 100% of them with a BK-positive result and 50% of them with a BK-negative result were correctly identified, both in terms of lineage and resistance, indicating the high sensitivity of the AmpliSeq technique. The SNPs used for lineage and drug-resistance identification can be found in Table S3.2.

## 4. Discussion

WGS is becoming affordable, with overall costs comparable to other tests currently in use to “all-in-one” perform the diagnosis of drug-resistant TB, cluster analysis and lineage characterisation. Nevertheless, WGS from the direct diagnostic specimen is not yet standardised. Sequencing from early MGIT requires positive cultures, whereas targeted sequencing can be performed from a specimen positive for *M. tuberculosis* with a consistent gain in time to information. In this work, we present a fast *M. tuberculosis* resistance diagnostic method from specimens based on the AmpliSeq methodology using Next-Generation Technology. With respect to the processing time of the technique, the DNA extraction can be conducted in 4–5 h, and the different AmpliSeq steps in one or two days, which means that in three days, a complete profile of the strains, both in terms of lineage and resistance profile, can be obtained. In addition, this technique is safer than methods based on bacterial cultures, which need a biosafety infrastructure that could be less strict when working with clinical samples, as the bacterial load is considerably lower. The cost per sample in our laboratory is around EUR 165, including the kits used for the DNA extraction (MolYsis 5 and AMPure XP magnetic beads), while the cost of WGS with Ion Torrent technology is around EUR 220. As a limitation, the samples have to be worked from eight to eight to optimize the price of library preparation, as the optimum number of samples to load on the chip in the Ion Torrent sequencer is 32; therefore, it is necessary to wait until the required number of samples is available to adjust expenses. The Deeplex^®^ Myc-TB commercial kit has been developed, showing accurate results and predicting strain resistance to 15 anti-TB drugs in 2 days [5]. Our AmpliSeq panel could be an alternative for laboratories with access to Ion Torrent platforms, as Deeplex works with Illumina technology. Our results are comparable to the ones obtained with Deeplex regarding the drug resistance (73–96% accuracy in Deeplex and around 90% in our panel) and are even better regarding the lineage identification (42–73% accuracy for some TB families in Deeplex against 95% in our panel). With respect to the timing to obtain results, both techniques are equivalent.

We have demonstrated that a minimum amount of DNA is required for this technique, allowing us to extract DNA directly from clinical samples instead of waiting for the bacterial growth cultures, which can take weeks. Some *M. tuberculosis* SNPs were detected in 63.2% of the BK-negative samples, and the lineage was correctly identified in 42.1% of them, including some with a negative result in the standard PCR performed after the DNA extraction, which highlights the sensitivity of the technique. However, AmpliSeq is not trustworthy for drug-resistance detection in samples with negative BK, in which the bacterial concentration is quite low. This was observed in sample 942, which was phenotypically resistant to isoniazid, but no SNP was detected in the related genes by AmpliSeq technology. Another advantage of the AmpliSeq technique is that it is a faster way to obtain a broad susceptibility profile of the strains. This is very important when starting treatment to avoid providing an incorrect treatment regimen that could potentially worsen the disease. Although there are some rapid-action kits to test resistance, such as *Xpert MTB/RIF* [9] for identifying rifampicin resistance, or MTBDR*sl* [10] for second-line drug resistance, there is no one kit that includes all potential varieties of drug resistance, both for first- and second-line treatments, as well as the full diversity of mutations that can cause resistance. Regarding the traditional susceptibility tests based on MGIT, although they work well for rifampicin, isoniazid, fluoroquinolones and aminoglycosides, they are less reliable for pyrazinamide and ethambutol [11], and can take weeks as they require bacterial growth. With the AmpliSeq panel, many examples of drug resistance can be studied all at once, and multiple targets of interest can be included at any time. Some discrepancies were found among the genotype and phenotype drug-resistance profiles, especially for pyrazinamide resistance. All possible scenarios were observed regarding pyrazinamide resistance: a strain with a confident SNP in *pncA* but that was phenotypically susceptible, two strains with no mutations in *pncA* but that were phenotypically resistant and a strain with an SNP not described as conferring resistance and that was phenotypically resistant. Two reasons can be proposed: the less reliable DST for pyrazinamide, and the likelihood that the resistant mechanisms of pyrazinamide are not yet completely understood. In this line, the Lys48Asn mutation in *pncA* (sample MTB-22) should be considered as conferring resistance, as we observed this fact. The same could be applied to the mutations in *katG* Ser315Arg (sample MTB-16) and *rpsL* Lys88Thr (sample MTB-20), as both strains were resistant to isoniazid and streptomycin, respectively. Regarding sample MTB-20, some mutations in the *gid* gene could explain the streptomycin resistance, as well as for sample 120 (phenotypically resistant to streptomycin but without any mutation in the *rrs* or *rpsL* genes) and sample MTB-22 (sequenced with the first version of the panel in which *rrs* was not included). However, the *gid* gene is not included as a target in our AmpliSeq panels. One limitation of our panel is the lack of some genes associated with resistance, the majority of them being for second-line and recently incorporated drugs. That could be the reason why we did not detect ethionamide resistance in sample MTB-14. Another limitation involves streptomycin resistance in clinical samples, as the *rrs* gene is incorrectly sequenced, with several SNPs, which could be due to the fact that this gene has conserved regions among bacteria [12,13], and in the clinical samples, the presence of DNA not belonging to *M. tuberculosis* could interfere with the alignment. We observed incorrect sequencing of *rrs* only in clinical samples, not in DNA derived from cultures, affecting 26 out of 38 samples analysed with the second version of the panel, the one that contained the *rrs* gene as a target. Based on this, the panel is not reliable for streptomycin resistance when applied to DNA extracted directly from clinical samples. Sample MTB-10 was amikacin-resistant in the DST, but the only mutation found to be possibly implicated in the resistance was in the *rrs* gene, although it was not described as conferring amikacin resistance, but rather streptomycin resistance. It is likely that this mutation is also implicated in the amikacin resistance of this strain. Regarding strain MTB-21, with a confident SNP in *embB* but susceptible to ethambutol in the DST, other authors have also found this mutation in susceptible isolates as well as in resistant isolates [3,14]. On the other hand, AmpliSeq identified pyrazinamide and fluoroquinolone resistance in strains for which the DST was not carried out, as normally, only first-line drugs are tested.

Resistant strains have been described to have reduced fitness [15]. Trying to understand this, some compensatory mutations have been observed for rifampicin-resistant strains harbouring *rpoB* mutations in the *rpoA* and *rpoC* genes [16], both encoding other subunits of the RNA polymerase. In this sense, *rpoC* was added as a target in our second version of the panel, hypothesising that some of the mutations found in *rpoB* and *rpoC* in the strains analysed could be compensatory. There was a low prevalence of samples that presented resistance, which is a limitation to carrying out analyses of predictive value for the different drugs.

Our panels also identified lineage and family, which is useful for genotyping as it is faster than traditional genotyping techniques such as IS*6110*-RFLP, spoligotyping or Mycobacterial Interspersed Repetitive Units—Variable Number of Tandem Repeats (MIRU-VNTR) [17]. It is important to remark that some families have clinical relevance, for example the Beijing family, known for being hypervirulent [18] and with a high prevalence of multidrug resistance [19].

The main advantage of AmpliSeq is the low DNA concentration required to obtain information regarding the lineage and the drug resistance, allowing us to directly process clinical samples. Additionally, this technique is somewhat cheaper than WGS, and a lower volume of computer data is generated, which could be an advantage. In addition, AmpliSeq results could easily be managed by untrained professionals, such as clinicians. Thanks to the designed algorithm, the data are shown automatically with the significance of each SNP, so the interpretation of the results is fast and automatic.

## Figures and Tables

**Table 1:** Characteristics of the samples and DNA concentration of clinical samples (after the extraction) and bacterial cultures (diluted from the original stock) used in the study. Fifty-five of the samples were analysed using the first version of the kit and fifty-six samples with the second version. LOW means that the concentration was below the detection limit of Qubit (0.1 ng).

Sample	ng/μL	Kind of Sample	BK	PCR	Sample	ng/μL	Kind of Sample
96	LOW	Sputum	+	Yes	MTB-1	12.5	Bacterial culture
91	LOW	Sputum	+	Yes	MTB-2	37.6	Bacterial culture
955	8.14	Sputum	+	Yes	MTB-3	23.9	Bacterial culture
52	2.28	Sputum	+	Yes	MTB-4	40.6	Bacterial culture
344	0.488	Sputum	+	Yes	MTB-5	55.4	Bacterial culture
692	0.598	Sputum	+	Yes	MTB-6	43	Bacterial culture
785	3.12	Sputum	+	Yes	MTB-7	11.2	Bacterial culture
532	0.162	Sputum	−	Yes	MTB-8	326	Bacterial culture
952	6	Sputum	+	Yes	MTB-9	35.8	Bacterial culture
684	8.5	Sputum	+	Yes	MTB-10	8.04	Bacterial culture
879	LOW	Pleural fluid	+	Yes	MTB-11	9.98	Bacterial culture
635	2.52	Biopsy	+	Yes	MTB-12	7.74	Bacterial culture
942	LOW	Biopsy	−	No	MTB-13	6.56	Bacterial culture
217	LOW	Sputum	+	Yes	MTB-14	2.66	Bacterial culture
659	5	Sputum	+	Yes	MTB-15	2	Bacterial culture
388	2.18	Sputum	+	Yes	MTB-16	1.56	Bacterial culture
658	LOW	Sputum	+	Yes	MTB-17	20.6	Bacterial culture
275	LOW	Sputum	+	Yes	MTB-18	11.8	Bacterial culture
698	5.72	Sputum	+	Yes	MTB-19	51.6	Bacterial culture
052	LOW	Sputum	+	Yes	MTB-20	2.86	Bacterial culture
542	1.67	Sputum	+	Yes	MTB-21	17.6	Bacterial culture
315	0.68	Sputum	+	Yes	MTB-22	10.3	Bacterial culture
40	LOW	Sputum	+	Yes	MTB-23	20.7	Bacterial culture
988	0.254	Sputum	−	Yes	MTB-24	8.4	Bacterial culture
212	0.368	Sputum	+	Yes	MTB-25	2.46	Bacterial culture
140	LOW	Sputum	+	Yes	MTB-26	40	Bacterial culture
381	LOW	Sputum	+	Yes	MTB-27	23	Bacterial culture
786	LOW	Sputum	+	Yes	MTB-28	2.96	Bacterial culture
644	LOW	Sputum	+	Yes	MTB-29	6.2	Bacterial culture
912	0.482	Sputum	+	Yes	MTB-30	43	Bacterial culture
120	LOW	Sputum	+	Yes	MTB-31	4.82	Bacterial culture
270	LOW	Biopsy	-	Yes	MTB-32	15.1	Bacterial culture
178	0.422	Sputum	+	Yes	MTB-33	472	Bacterial culture
537	0.454	Sputum	−	Yes	MTB-34	14.8	Bacterial culture
916	0.138	Sputum	+	Yes	MTB-35	139	Bacterial culture
521	0.122	Sputum	-	Yes	MTB-36	32.2	Bacterial culture
736	1.92	Sputum	+	Yes	MTB-37	60.6	Bacterial culture
69	LOW	Sputum	+	Yes	MTB-38	111	Bacterial culture
263	0.19	Sputum	+	Yes	MTB-39	21.4	Bacterial culture
453	7.22	Sputum	+	Yes	MTB-40	104	Bacterial culture
163	0.106	Aspirate	+	No	MTB-41	70.2	Bacterial culture
667	0.162	Aspirate	+	Yes	MTB-42	48.2	Bacterial culture
882	0.328	Sputum	+	Yes	MTB-43	3.92	Bacterial culture
007	LOW	Sputum	+	No	MTB-44	8.08	Bacterial culture
640	LOW	Lavage	−	No	MTB-45	108.7	Bacterial culture
716	LOW	Aspirate	−	No	MTB-46	6.12	Bacterial culture
907	LOW	Pleural fluid	−	No	MTB-47	8.42	Bacterial culture
100	0.964	Sputum	−	No	MTB-48	6.24	Bacterial culture
561	LOW	Adeno puncture	−	No	MTB-49	2	Bacterial culture
327	LOW	Aspirate	−	No	MTB-50	14.3	Bacterial culture
343	LOW	Aspirate	−	No	MTB-51	0.744	Bacterial culture
590	0.114	Sputum	−	No	MTB-52	1.292	Bacterial culture
169	LOW	Pleural fluid	−	No			
273	1.54	Sputum	+	Yes			
884	0.152	Lavage	−	No			
295	0.202	Sputum	−	No			
318	LOW	Aspirate	+	No			
473	0.84	Sputum	−	No			
366	LOW	Pleural fluid	−	No			

## Data Availability

Not applicable.

## Supplementary Materials

The following supporting information can be downloaded at: https://www.mdpi.com/article/10.3390/microorganisms11061467/s1, Table S1: Amplicons of the first and second version of the panels (IAD88259 and IAD137392); Table S2: AmpliSeq results for the 111 samples analysed. Table S3.1: Points of interest for identification of lineage/family; Table S3.2: Points of interest for identification of antimicrobial resistance.
